# Dose-escalated icotinib in frail, older adults patients with *EGFR*-mutant lung adenocarcinoma: real-world efficacy and safety in a high-risk cohort

**DOI:** 10.3389/fonc.2025.1700686

**Published:** 2025-12-10

**Authors:** Junhui Wang, Jian Wang, Jianxin Chen

**Affiliations:** 1 Department of Radiation. The Quzhou Affiliated Hospital of Wenzhou Medical University, Quzhou People′s Hospital, Quzhou, Zhejiang, China; 2 Department of Gastroenterology, Jiaxing Second Hospital, Jiaxing, Zhejiang, China; 3 Department of Education, International Word. The Quzhou Affiliated Hospital of Wenzhou Medical University, Quzhou People′s Hospital, Quzhou, Zhejiang, China

**Keywords:** dose escalation, icotinib, older adults patients, EGFR-mutant lung adenocarcinoma, real-world study

## Abstract

**Background:**

The therapeutic potential of dose-escalated EGFR-TKIs in high-risk *EGFR*-mutant lung adenocarcinoma patients remains underexplored. This real-world study evaluated first-line double-dose icotinib (750 mg/day) in frail, older adults patients with poor performance status (ECOG PS 2).

**Methods:**

A single-center retrospective cohort analysis included 17 treatment-naïve patients with locally advanced/metastatic *EGFR*-mutant (ex19del/L858R) lung adenocarcinoma. All received icotinib 250 mg TID. Primary endpoints: objective response rate (ORR), disease control rate (DCR), progression-free survival (PFS), overall survival (OS), and safety. Subgroup analyses assessed metastatic burden, mutation subtypes, and systemic inflammation biomarkers.

**Results:**

The study cohort, characterized by high-risk features including a median age of 73 years, poor performance status (ECOG PS 2 in 88.2% of patients), baseline brain metastases (41.2%), and multi-organ involvement (≥2 metastatic sites in 47.1%), demonstrated clinically significant antitumor activity. The objective response rate (ORR) was 52.9% (95% confidence interval [CI]: 27.8%–77.0%), while the disease control rate (DCR) reached 94.1% (95% CI: 71.3%–99.9%). Survival analyses revealed a median progression-free survival (PFS) of 14.6 months (95% CI: 2.63–26.58) and a median overall survival (OS) of 30.9 months (95% CI: 20.63–41.18). Notably, molecular stratification identified a significant survival advantage for *EGFR* L858R-mutant patients over those with exon 19 deletions (ex19del), with a hazard ratio (HR) for OS of 0.17 (95% CI: 0.05–0.65; p<0.01). Conversely, patients with ≥2 metastatic organs exhibited inferior PFS outcomes (HR = 3.18, 95% CI: 1.01–10.06; p=0.049). The safety profile remained favorable, with only one grade 3 rash (5.9%) reported among treatment-emergent adverse events, and no therapy discontinuations due to toxicity were observed throughout the study period.

**Conclusion:**

Double-dose icotinib shows robust efficacy and manageable toxicity in high-risk older adults patients, with clinically meaningful PFS/OS. The unexpected OS benefit in L858R mutants warrants validation but suggests a dose-dependent advantage. Metastatic burden remains a key prognostic factor. These findings support dose escalation as a viable strategy for frail populations with limited access to next-generation TKIs.

## Introduction

Lung adenocarcinoma remains the predominant histological subtype of non-small cell lung cancer (NSCLC), with epidermal growth factor receptor (*EGFR*) sensitizing mutations (primarily exon 19 deletions and exon 21 L858R point mutations) identified as critical oncogenic drivers in approximately 10-15% of Caucasian and 40-50% of Asian patients (1, 2). For patients harboring these mutations, EGFR tyrosine kinase inhibitors (EGFR-TKIs) have revolutionized first-line treatment, significantly improving progression-free survival (PFS) and overall survival (OS) compared to conventional platinum-based chemotherapy (3, 4).

Icotinib, a first-generation EGFR-TKI developed in China, has demonstrated robust efficacy and favorable tolerability in multiple phase III trials. The landmark ICOGEN study established icotinib (125 mg three times daily) as non-inferior to gefitinib in terms of PFS, with a comparable safety profile in patients with advanced NSCLC pretreated with chemotherapy (5). Subsequent real-world studies and the CONVINCE trial further supported its efficacy as first-line therapy for *EGFR*-mutant NSCLC (6–8). Despite these advances, acquired resistance inevitably develops, leading to disease progression. Furthermore, a subset of patients, particularly those with high tumor burden, brain metastases, or poor performance status, may exhibit suboptimal initial responses or rapid progression on standard-dose EGFR-TKI therapy (9).

Therapeutic dose escalation represents a potential strategy to overcome pharmacokinetic limitations or enhance target inhibition in challenging scenarios. Preclinical and early clinical pharmacokinetic (PK) studies indicate that icotinib exhibits dose-proportional increases in exposure within a certain range (10–13). A prior phase I dose-escalation study demonstrated that a higher dose of icotinib (250 mg TID, total daily dose 750 mg) was feasible and achieved enhanced plasma concentrations, suggesting potential for improved efficacy without a proportionate increase in severe adverse events (11). Critically, the phase II INCREASE trial provided the first prospective clinical evidence supporting this approach. This multicenter study specifically evaluated double-dose icotinib (250 mg TID) versus standard dose (125 mg three times daily) in *EGFR* L858R-mutant advanced NSCLC patients. Results demonstrated significantly improved median PFS and objective response rate with the double-dose regimen, particularly pronounced in those with baseline brain metastases, while maintaining manageable toxicity (14). However, comprehensive real-world evidence specifically evaluating the efficacy and safety of this double-dose icotinib regimen (750 mg/day) as first-line therapy for patients with locally advanced or metastatic *EGFR*-mutant lung adenocarcinoma, especially in broader molecular subgroups (including both 19del and L858R) and high-risk populations (e.g., older adults, poor PS), remains limited.

This study aimed to address this evidence gap by conducting a real-world, retrospective cohort analysis at Quzhou People’s Hospital. We evaluated the efficacy (objective response rate [ORR], disease control rate [DCR], PFS, OS) and safety profile of double-dose icotinib (250 mg TID) in a cohort of patients with *EGFR* 19del or L858R mutant lung adenocarcinoma, focusing particularly on outcomes in clinically relevant subgroups and exploring the prognostic value of systemic inflammation biomarkers.

## Methods

### Study design and setting

This was a real-world, single-center, retrospective cohort study conducted at Quzhou People’s Hospital, China. The study aimed to evaluate the efficacy and safety of double-dose icotinib in patients with locally advanced or metastatic lung adenocarcinoma harboring *EGFR* sensitizing mutations. We reviewed electronic medical records from January 2018 to December 2023, encompassing all eligible patients who received the intervention during this period. The retrospective design allowed for comprehensive data extraction without prospective intervention, aligning with real-world evidence generation for clinical practice.

### Patient population

Patients were included if they met the following criteria: (1) histologically or cytologically confirmed diagnosis of lung adenocarcinoma; (2) locally advanced (stage III) or metastatic (stage IV) disease, as staged according to the 8th edition of the TNM classification by the International Association for the Study of Lung Cancer (IASLC); (3) documented *EGFR* sensitizing mutations, specifically exon 19 deletion (19del) or exon 21 L858R point mutation, detected via next-generation sequencing (NGS) of tumor tissue or plasma circulating tumor DNA; (4) treatment with double-dose icotinib as first-line therapy; and (5) availability of complete clinical and laboratory data for analysis. Exclusion criteria included: (1) presence of other *EGFR* mutations (e.g., T790M or exon 20 insertions), (2) concurrent active malignancies, or (3) incomplete follow-up records. A total of 17 consecutive patients were enrolled based on these criteria. Baseline demographic and clinical characteristics were systematically collected, including age, gender, smoking status, Eastern Cooperative Oncology Group Performance Status (ECOG PS), tumor stage, brain metastasis status, number of metastatic organs, and mutation subtype, as detailed in the results.

### Treatment protocol

All patients received oral icotinib at a double dose of 250 mg TID (total daily dose of 750 mg), consistent with prior pharmacokinetic studies demonstrating enhanced bioavailability at this dosage. Treatment initiation occurred after confirmation of *EGFR* mutation status, and therapy was administered continuously until disease progression, unacceptable toxicity, or patient withdrawal. Dose reductions or interruptions were permitted for management of adverse events, based on investigator discretion and institutional guidelines. Concomitant therapies, such as pemetrexed or bevacizumab combinations, were recorded but not standardized, reflecting real-world variability in clinical practice.

All patients received oral icotinib at a double dose of 250 mg TID (total daily dose of 750 mg). Treatment initiation occurred after confirmation of *EGFR* mutation status, and therapy was administered continuously until disease progression, unacceptable toxicity, or patient withdrawal. Dose reductions or interruptions were permitted for management of adverse events, based on investigator discretion and institutional guidelines. Concomitant therapies, such as pemetrexed or bevacizumab combinations, were recorded but not standardized, reflecting real-world variability in clinical practice.

### Data collection and variables

Data were extracted from electronic health records by trained oncology staff using a standardized case report form. Baseline variables included: Demographic factors: age, gender, and smoking status (categorized as nonsmoker or former/current smoker). Clinical characteristics: ECOG PS (dichotomized as 0–1 or 2), tumor stage, presence of brain metastases, and number of metastatic organs (categorized as <2 or ≥2). Molecular pathology: *EGFR* mutation subtype (19del or L858R). Systemic inflammation biomarkers: Pre-treatment blood samples were analyzed for complete blood counts and serum markers. Ratios were calculated as follows: neutrophil-to-lymphocyte ratio (NLR), platelet-to-lymphocyte ratio (PLR), lymphocyte-to-monocyte ratio (LMR), platelet-to-albumin ratio (PAR), systemic immune-inflammation index (SII, calculated as platelets × neutrophils/lymphocytes), neutrophil-to-platelet ratio (NPR), C-reactive protein-to-albumin ratio (CAR), C-reactive protein-to-lymphocyte ratio (CLR), C-reactive protein (CRP), and lactate dehydrogenase (LDH). These biomarkers were measured at baseline and periodically during follow-up to assess correlations with outcomes.

### Outcome measures

Tumor response was assessed based on the change in the sum of diameters of target lesions, as defined by RECIST v1.1. Target lesions were selected as measurable lesions (with a minimum size of 10 mm in the longest diameter for non-nodal lesions or short axis for nodal lesions), with up to 5 target lesions per organ and 10 lesions in total. The best percentage change in the sum of diameters was calculated from baseline to the smallest sum recorded during treatment. Efficacy endpoints were evaluated per Response Evaluation Criteria in Solid Tumors (RECIST) version 1.1. Short-term efficacy: Objective response rate (ORR) was defined as the proportion of patients achieving complete response (CR) or partial response (PR). Disease control rate (DCR) included CR, PR, and stable disease (SD). Tumor response assessments were performed via computed tomography (CT) scans every 8–12 weeks, with best tumor size changes visually summarized. ​Long-term efficacy: Progression-free survival (PFS) was measured from treatment initiation to disease progression or death from any cause. Overall survival (OS) was defined as the time from treatment start to death or last follow-up. Survival curves were generated to visualize these outcomes. Safety profile: Adverse events (AEs) were graded according to the National Cancer Institute Common Terminology Criteria for Adverse Events (CTCAE) version 5.0, with documentation of all-grade and grade 3–4 events. AEs of special interest included hematological toxicities (e.g., anemia, thrombocytopenia), fatigue, rash, neutropenia, and liver impairment.

### Statistical analysis

Descriptive statistics summarized baseline characteristics, with continuous variables expressed as median (range) or mean ± standard deviation (SD), and categorical variables as frequencies and percentages. Efficacy analyses included as follows. Calculation of ORR and DCR with 95% confidence intervals (CIs) using the Clopper-Pearson exact method. Survival analyses: Median PFS and OS were estimated using the Kaplan-Meier method, with 95% CIs derived from log-log transformations. Univariate Cox proportional hazards regression models identified prognostic factors for OS, with hazard ratios (HRs) and 95% CIs reported. Subgroup analyses: Pre-specified subgroups based on clinical features (e.g., number of metastatic organs) and molecular characteristics (e.g., *EGFR* mutation subtype) were analyzed for PFS and OS using log-rank tests and Kaplan-Meier curves to explore heterogeneity in treatment response. Safety data were summarized descriptively. All analyses were performed using SPSS version 26.0 (IBM Corp., Armonk, NY), with a two-sided significance level of p < 0.05 considered statistically significant.

### Ethical considerations

The study protocol was approved by the Ethics Committee of Quzhou People’s Hospital (approval number: QZPH-IRB-2022-007). Given the retrospective nature, informed consent was waived in accordance with local regulatory guidelines, but patient data were anonymized to ensure confidentiality and compliance with the Declaration of Helsinki.

## Results

### Patient characteristics

A total of 17 patients with locally advanced or metastatic *EGFR*-mutant lung adenocarcinoma were retrospectively enrolled. Baseline characteristics are summarized in **Table 1**. The median age was 73 years (range: 61–87), with 82.4% (n=14) aged ≥65 years. Slightly more females (52.9%, n=9) were included. Most patients had stage IV disease (76.5%, n=13), and 41.2% (n=7) presented with brain metastases. The cohort predominantly comprised poor-performance status patients (ECOG PS 2: 88.2%, n=15) and non-smokers (88.2%, n=15). Regarding treatment, 76.5% (n=13) received icotinib monotherapy, while 23.5% (n=4) received combination regimens (pemetrexed or bevacizumab). Mutation subtypes were balanced (19del: 41.2% [n=7]; L858R: 58.8% [n=10]). Systemic inflammation markers exhibited considerable heterogeneity (mean ± SD): NLR (5.86 ± 4.56), PLR (193.12 ± 101.56), SII (1278.45 ± 1338.42), and LDH (268.12 ± 100.05 U/L).

**Table 1:** Baseline characteristics.

Baseline characteristics	All patients (n = 17)
Age (years), n (%)	
Median (range)	73 (61-87)
≥65	14 (82.4)
<65	3 (17.6)
Gender, n (%)	
Male	8 (47.1)
Female	9 (52.9)
Brain metastasis, n (%)	7 (41.2)
Smoking status, n (%)	
Nonsmoker	15 (88.2)
Former smoker/smoker	2 (11.8)
Tumor staging, n (%)	
Stage III	4 (23.5)
Stage IV	13 (76.5)
Number of metastatic organs, n (%)	
≥2	8 (47.1)
<2	9 (52.9)
ECOG PS, n (%)	
0–1	2 (11.8)
2	15 (88.2)
Treatment regimen, n (%)	
Monotherapy	13 (76.5)
Pemetrexed combination	1 (5.9)
Bevacizumab combination	3 (17.6)
Mutation subtype	
*EGFR* 19del	7 (41.2)
*EGFR* L858R	10 (58.8)
Icotinib, median (IQR)	10 (4-20)
level of systemic inflammation, (mean ± *SD*)	
NLR	5.86 ± 4.56
PLR	193.12 ± 101.56
LMR	2.45 ± 1.19
PAR	5.78 ± 2.12
SII	1278.45 ± 1338.42
NPR	0.03 ± 0.01
CAR	0.69 ± 1.00
CLR	25.76 ± 43.71
CRP (mg/L)	22.65 ± 31.58
LDH (U/L)	268.12 ± 100.05

NLR, Neutrophil-to-Lymphocyte Ratio; PLR, Platelet-to-Lymphocyte Ratio; LMR, Lymphocyte-to-Monocyte Ratio; PAR, Platelet-to-Albumin Ratio; SII, Systemic Immune-Inflammation Index (Platelets × Neutrophils/Lymphocytes); NPR, Neutrophil-to-Platelet Ratio; CAR, C-reactive Protein-to-Albumin Ratio; CLR, C-reactive Protein-to-Lymphocyte Ratio; CRP, C-reactive Protein; LDH, Lactate Dehydrogenase.

### Treatment efficacy

**Figure 1:**
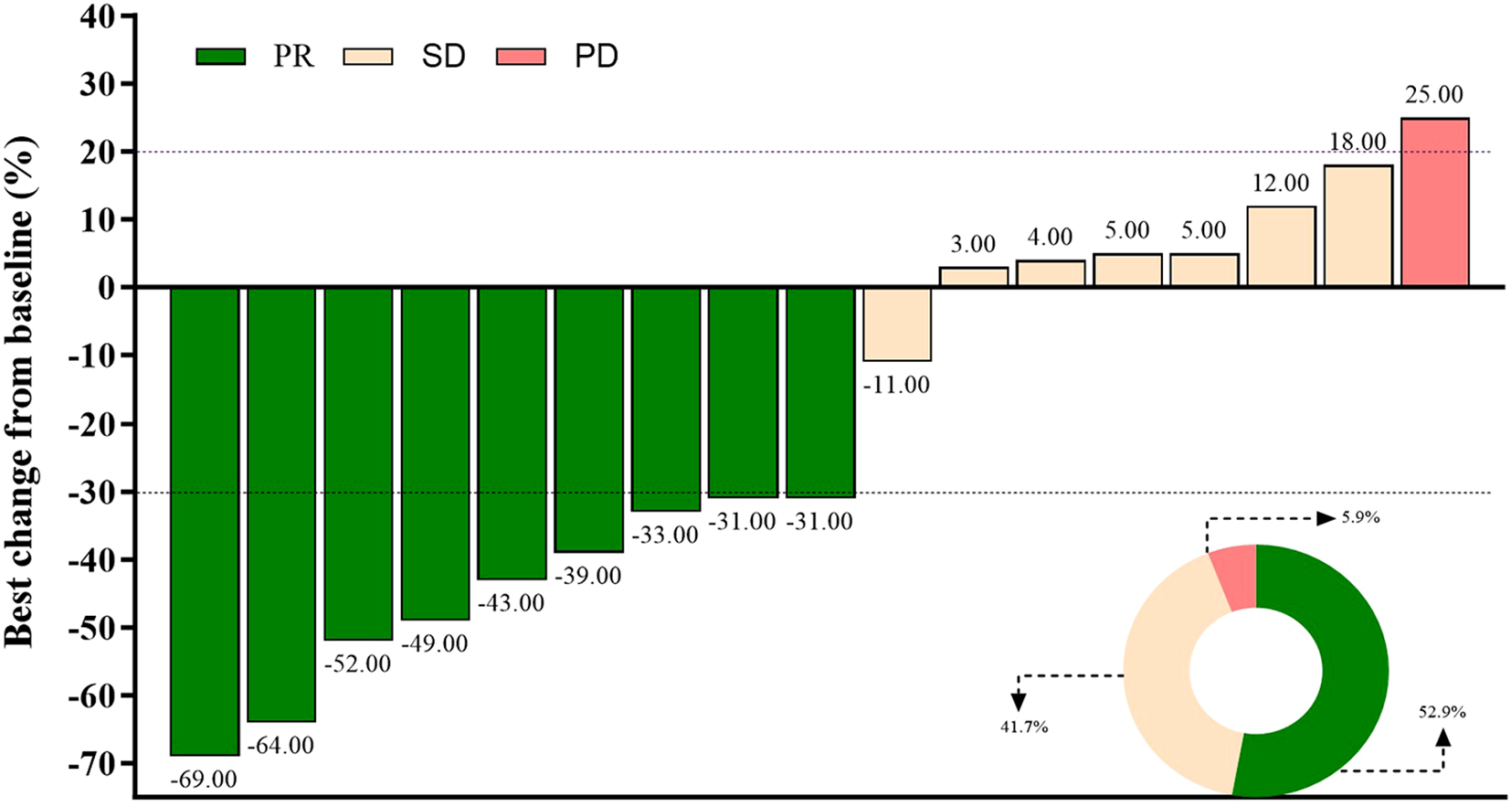
Best percentage change in the sum of diameters of target lesions per RECIST v1.1. Partial response (PR, green), stable disease (SD, beige), and progressive disease (PD, pink) are shown for each patient.

Short-term efficacy outcomes are detailed in **Table 2**. The objective response rate (ORR) was 52.9% (95% CI: 27.8%–77.0%), with all responses classified as partial responses (PR, n=9). Stable disease (SD) was observed in 41.2% (n=7), resulting in a disease control rate (DCR) of 94.1% (95% CI: 71.3%–99.9%). Only one patient (5.9%) experienced progressive disease (PD). Tumor size changes for individual patients are depicted in **Figure 1**, visually highlighting the distribution of PR (green), SD (beige), and PD (pink).

**Table 2:** Efficacy of double icotinib in NSCLC patients (n = 17).

Efficacy	All patients (n = 17)
Complete response (%)	0
Partial response (%)	9 (52.9)
Stable disease (%)	7 (41.2)
Progressive disease (%)	1 (5.9)
Objective response rate (%, CR, PR)	9 (52.9), 95% CI:27.8% ~ 77.0%
Disease control rate (%, CR, PR, SD)	16 (94.1), 95% CI:71.3% ~ 99.9%
Median progression-free survival (months, 95% CI)	14.6 (2.63, 26.58)
Median overall survival (months, 95% CI)	30.9 (20.63,41.18)

**Figure 2:**
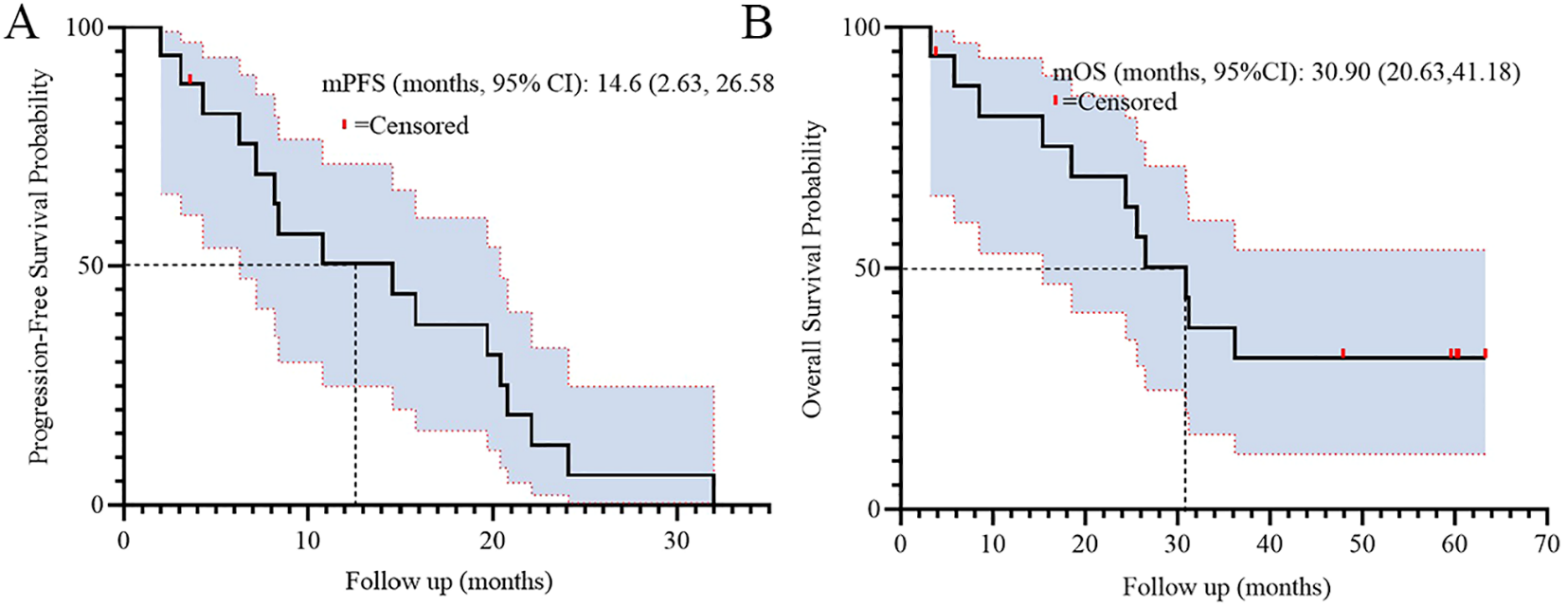
Kaplan-Meier survival curves of PFS **(A)** and OS **(B)** among all patients.

For long-term outcomes, the median progression-free survival (PFS) was 14.6 months (95% CI: 2.63–26.58), and median overall survival (OS) was 30.9 months (95% CI: 20.63–41.18). **Figure 2** illustrates the Kaplan-Meier curves for PFS (A) and OS (B), demonstrating sustained clinical benefit in the cohort.

### Prognostic analyses

**Figure 3:**
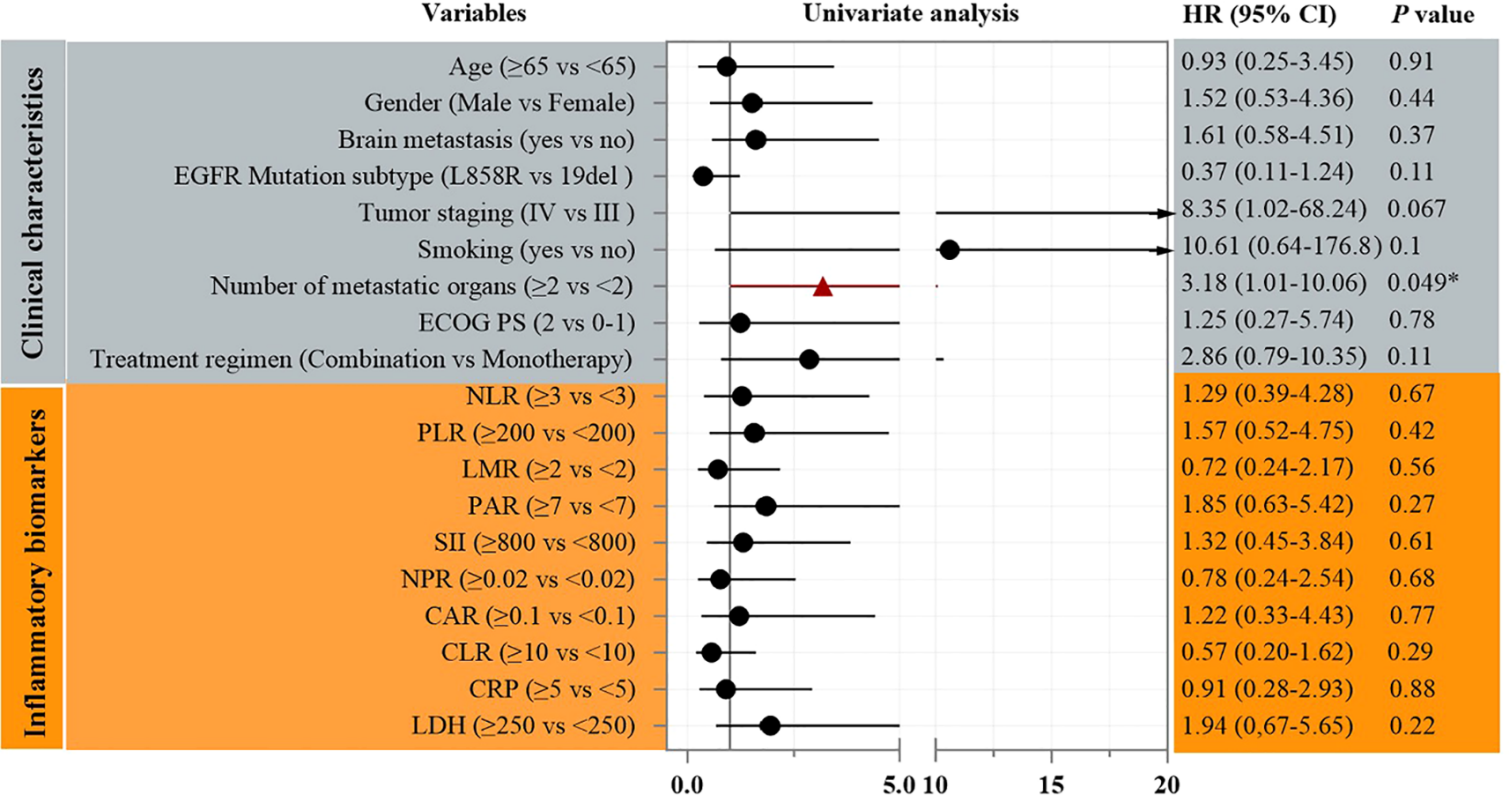
Univariate analysis of prognostic factors for PFS.

**Figure 4:**
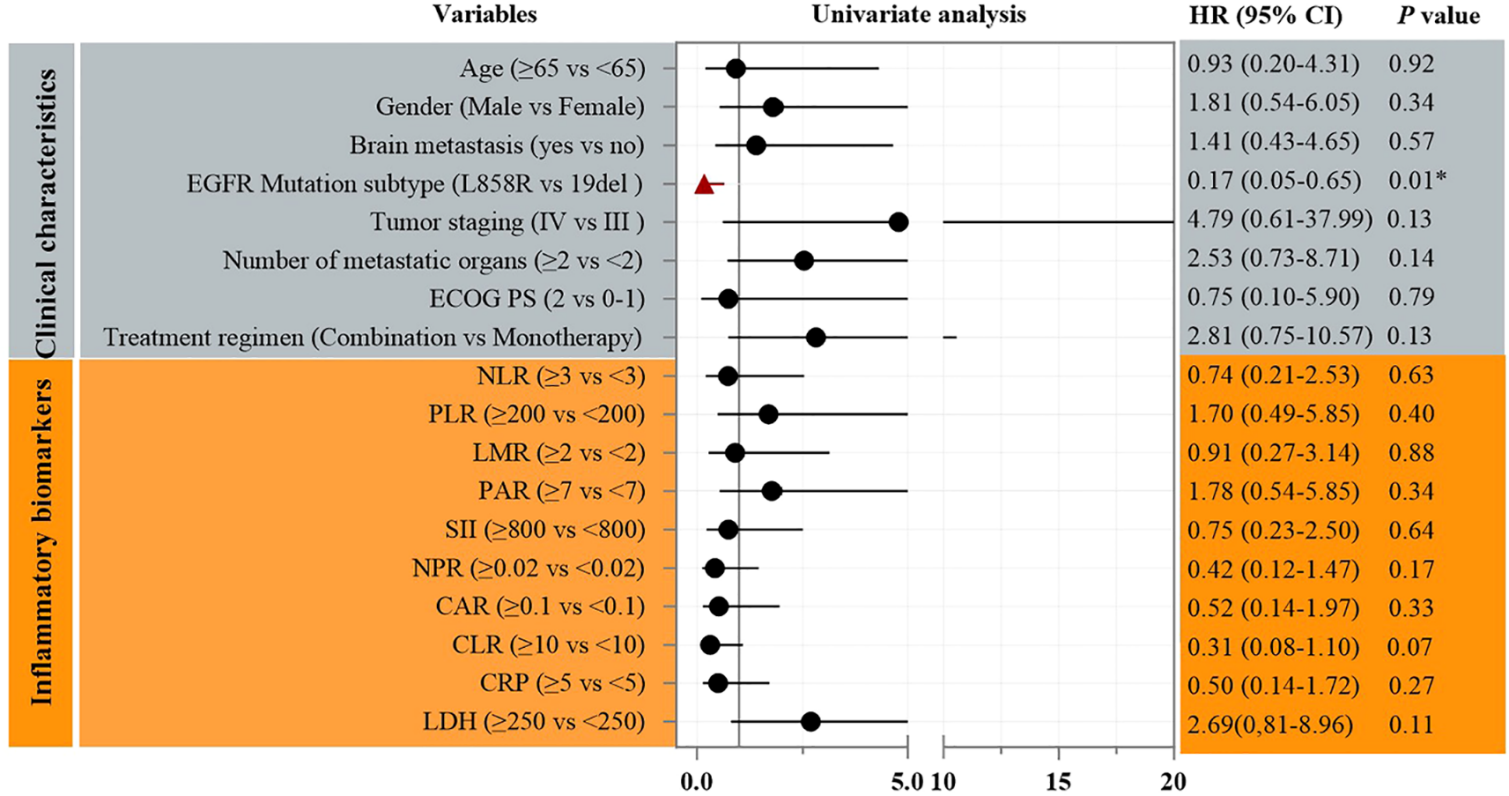
Univariate analysis of prognostic factors for OS.

Univariate Cox analysis identified the number of metastatic organs as a significant prognostic factor for PFS (HR = 3.18, 95% CI: 1.01–10.06; p=0.049, **Figure 3**). Patients with ≥2 metastatic sites had inferior survival compared to those with <2 sites. In addition, Univariate Cox analysis also identified the *EGFR* mutation subtype as a significant prognostic factor for OS (HR = 0.17, 95% CI: 0.05–0.65; p<0.01, **Figure 4**). Patients with *EGFR* 19 deletion had inferior survival compared to those harboring *EGFR* 21 L858R mutation. Other variables, including age, gender, brain metastasis, staging, or PS status did not reach statistical significance.

### Subgroup analyses

**Figure 5:**
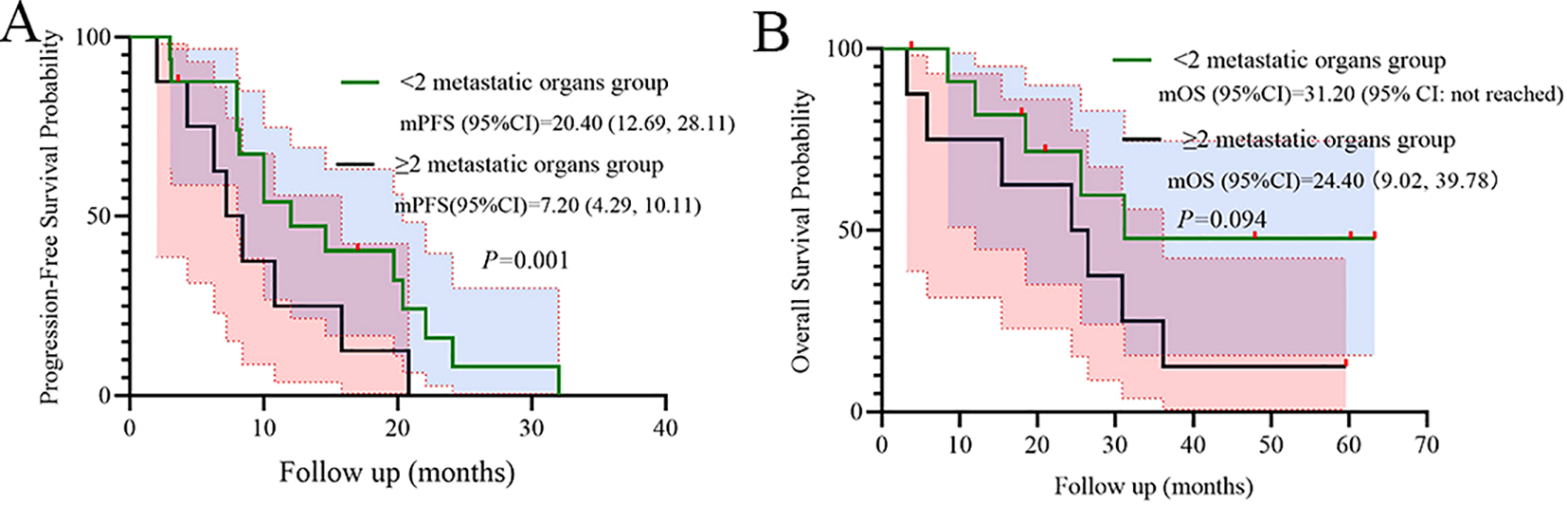
Subgroup analysis of Metastatic organs as prognostic factors for PFS **(A)** and OS **(B)**.

Metastatic burden significantly stratified PFS (p=0.001), while a numerical trend was observed for OS (p=0.094), indicating poorer outcomes in the subgroup with ≥2 metastatic organs (**Figure 5**).

**Figure 6:**
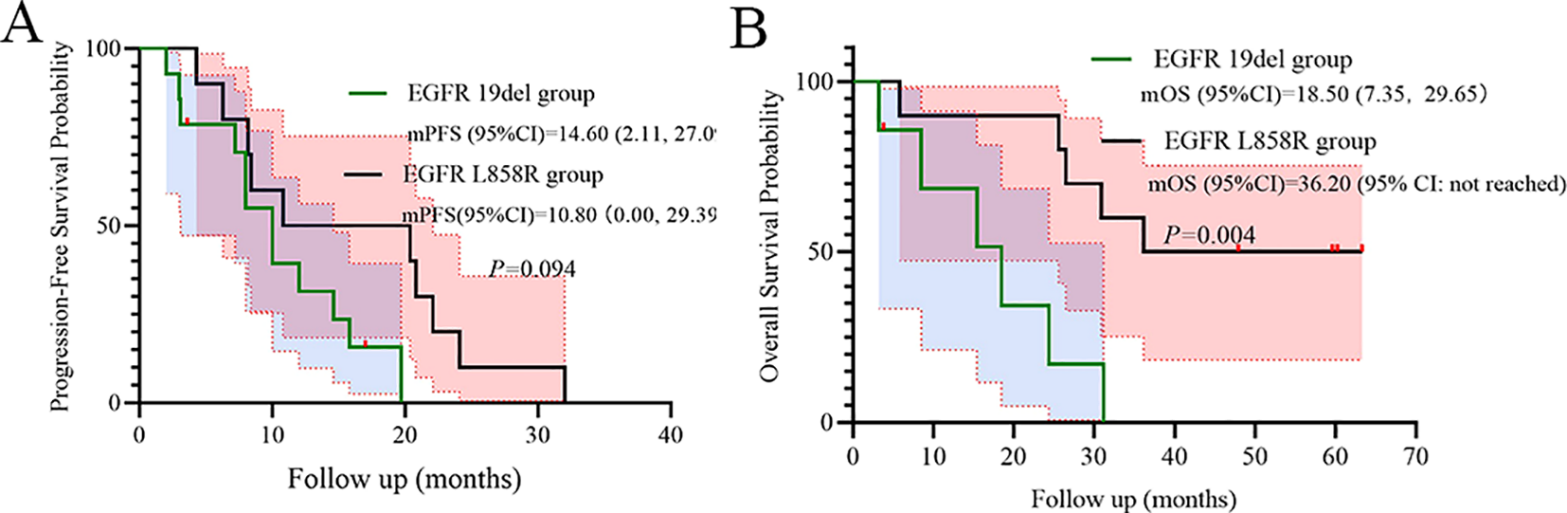
Subgroup analysis of Mutation subtype as prognostic factors for PFS **(A)** and OS **(B)**.

Mutation subtype trends favored *EGFR* 19del over L858R for PFS (HR = 0.41, p=0.094, **Figure 6A**). However, *EGFR* L858R mutation was shown significantly superior to *EGFR* 19del for OS (p=0.004, **Figure 6B**).

### Safety profile

Adverse events (AEs) were predominantly low-grade (**Table 3**). The most common all-grade AEs were fatigue (35.4%, n=6), anemia (29.4%, n=5), and rash (29.4%, n=5). Only one grade 3–4 AE was reported (rash: 5.9%, n=1). No treatment-related deaths or discontinuations due to toxicity occurred.

**Table 3:** Adverse events.

Adverse events	Grade 1–2, n (%)	Grade 3–4, n (%)
Anemia	5 (29.4)	0
Thrombocytopenia	2 (11.8)	0
Fatigue	6 (35.4)	0
Rash	4 (23.6)	1 (5.9)
Neutropenia	4(23.6)	0
Liver impairment	2 (11.8)	0

## Discussion

This real-world, retrospective cohort study provides valuable clinical evidence supporting the efficacy and manageable safety of first-line double-dose icotinib (750 mg/day) in patients with advanced *EGFR*-mutant lung adenocarcinoma, particularly enriching the existing literature by focusing on a challenging population characterized by advanced age, poor performance status (ECOG PS 2 predominance), and high metastatic burden. Our findings corroborate and extend the prospective evidence from the phase II INCREASE trial, demonstrating robust activity in a real-world setting where standard-dose EGFR-TKIs might exhibit suboptimal outcomes.

The observed ORR of 52.9% and DCR of 94.1% underscore substantial anti-tumor activity, aligning closely with the INCREASE trial’s reported ORR while considering the notably poorer baseline characteristics of our cohort (median age 73 years, 88.2% ECOG PS 2, 41.2% brain metastases) compared to typical clinical trial populations (14). The median PFS of 14.6 months (95% CI: 2.63–26.58) and median OS of 30.9 months (95% CI: 20.63–41.18) are clinically meaningful, especially given that 76.5% had stage IV disease and nearly half (47.1%) presented with ≥2 metastatic organs. These results suggest that dose escalation can overcome pharmacokinetic limitations or suboptimal target inhibition in high-risk subgroups, consistent with preclinical PK data showing dose-proportional exposure increases with icotinib (12, 13). The feasibility of this strategy in a predominantly older adults, frail population is particularly significant for regions like Asia where *EGFR* mutations are prevalent and access to newer-generation TKIs may be limited.

The PFS benefit observed here (14.6 months) favorably compares to historical real-world data for standard-dose first-generation TKIs (gefitinib/erlotinib: median PFS ~10 to 13 months) (15–17), and approaches the efficacy reported for osimertinib in treatment-naïve patients (median PFS 18.9 months in FLAURA) (18). Notably, the PFS in our study exceeded that reported in the CONVINCE trial for standard-dose icotinib in similar mutation carriers (median PFS 11.2 months for icotinib vs. 7.9 months for chemotherapy) (6). This reinforces the hypothesis that dose intensification may enhance depth and duration of response, particularly relevant for patients with high tumor burden or compromised drug exposure.

A pivotal finding demanding careful interpretation is the significantly superior OS observed in patients harboring the L858R mutation compared to those with 19del (p=0.004, **Figure 6B**). This contrasts with numerous reports suggesting better outcomes for 19del with standard-dose first-generation TKIs (19–21). Several factors may contribute to this apparent paradox as below. Dose-dependent differential efficacy: The INCREASE trial specifically demonstrated a greater PFS benefit from double-dose icotinib in L858R patients compared to 19del (HR 0.75 for L858R vs. HR 1.02 for 19del) (14). Our OS data (HR = 0.17 for L858R vs. 19del) may reflect an amplification of this dose-dependent effect on long-term survival in L858R carriers. Higher plasma concentrations achievable with 750 mg/day might more effectively overcome potential differences in EGFR dimerization stability or signaling kinetics associated with L858R (22–24). High-risk cohort dynamics: Our cohort’s unique characteristics-high prevalence of poor PS (88.2%) and advanced age (median 73 yrs)-might unmask differential vulnerabilities. L858R tumors in frail patients could be more susceptible to profound EGFR inhibition, while 19del tumors, often associated with higher metastatic potential, might progress more aggressively in this compromised host environment despite dose escalation. Sample size limitations: The small cohort size (n=17) increases vulnerability to type I/II errors. While statistically significant (p<0.01), the HR confidence interval (0.05–0.65) is wide, urging cautious generalization.

The univariate analysis identifying ≥2 metastatic organs as a strong adverse prognostic factor for PFS (HR = 3.18, p=0.049, **Figure 3**) aligns with established literature (25–27). High metastatic burden reflects aggressive tumor biology and likely contributes to rapid acquired resistance via clonal heterogeneity or bypass track activation, which even enhanced EGFR inhibition may not fully suppress long-term. This reinforces the need for combinatorial strategies in such patients.

Although not reaching statistical significance in multivariate models, the baseline systemic inflammation markers (mean NLR 5.86 ± 4.56, PLR 193.12 ± 101.56, SII 1278.45 ± 1338.42) trended towards association with worse outcomes, consistent with extensive evidence linking inflammation to immunosuppression, angiogenesis, and treatment resistance in NSCLC (28, 29). The heterogeneity observed warrants future larger studies exploring these biomarkers as dynamic predictors during dose-escalated TKI therapy.

The compelling Phase III data for osimertinib, a third-generation EGFR-TKI, has undoubtedly established it as a standard first-line therapy for *EGFR*-mutant NSCLC in settings where it is accessible and affordable. Our findings regarding double-dose icotinib must therefore be contextualized within this current treatment landscape. We posit that double-dose icotinib retains significant relevance, primarily in resource-limited settings where the high cost of next-generation TKIs like osimertinib presents a substantial barrier. It serves as a viable and potentially more accessible therapeutic option. Furthermore, this strategy may be particularly suited for specific patient subgroups, such as those with the *EGFR* L858R mutation, where the INCREASE trial and our data suggest a pronounced benefit from dose escalation. Its role may also be considered for patients with baseline brain metastases who are ineligible for or cannot tolerate osimertinib, leveraging the enhanced CNS penetration suggested by higher dosing. The favorable tolerability profile of icotinib, even at double doses, makes it an attractive alternative for frail or older adults patients who may be susceptible to the different toxicity profile of newer agents. Future research directions should focus on direct comparative effectiveness studies between dose-escalated first-generation TKIs and osimertinib in these specific niches, and on exploring combinatorial strategies to overcome resistance mechanisms, particularly in patients with high metastatic burden.

The safety profile was favorable, mirroring prior reports for icotinib (5, 14). Predominantly grade 1–2 adverse events (fatigue 35.4%, rash 29.4%, anemia 29.4%) and only one grade 3 rash (5.9%) with no discontinuations confirm the tolerability of 750 mg/day even in this older adults, frail cohort. This contrasts with the higher rates of severe toxicities often seen with dose escalation of other TKIs (e.g., afatinib) (30, 31), highlighting icotinib’s unique therapeutic window.

While providing clinically relevant insights, this research has several limitations that warrant careful consideration. First, the relatively small sample size (n=17), though reflective of a highly specific high-risk population, inherently restricts the statistical power for robust subgroup analyses and multivariate assessments, potentially limiting the generalizability of the findings. This is compounded by the single-center, retrospective design, which may introduce selection bias and unmeasured confounding factors not accounted for in our analysis. Second, the real-world nature of the study led to heterogeneous assessment intervals for radiographic tumor evaluations (typically every 8–12 weeks), which, despite adherence to RECIST v1.1, are less frequent and standardized than those in prospective trials, potentially delaying the detection of disease progression and influencing the reported progression-free survival (PFS). Third, the retrospective capture of adverse events (AEs) is susceptible to underreporting, as it relies on electronic medical records rather than proactive, systematic patient inquiry; consequently, mild or moderate AEs may have been missed, likely resulting in a more favorable safety profile than would be observed in a controlled clinical trial setting. Furthermore, the absence of a standardized protocol for treatment beyond radiological progression introduces another potential confounding variable, although our analysis strictly defined PFS from initiation to first documented progression. Finally, the study did not systematically analyze acquired resistance mechanisms or serially monitor inflammatory biomarkers, which could have provided deeper insights into the dynamics of treatment response and resistance. These limitations underscore the necessity for cautious interpretation of the results and highlight the critical importance of validating these conclusions through larger, prospective, ideally multi-center studies in the future.

In this real-world cohort of predominantly older adults and poor-performance status patients with advanced *EGFR*-mutant lung adenocarcinoma, first-line double-dose icotinib (750 mg/day) demonstrated clinically meaningful efficacy (median PFS 14.6 months, median OS 30.9 months) and a favorable safety profile. The significant OS advantage observed in L858R-mutant patients versus 19del requires validation in larger cohorts but aligns with the differential dose-response suggested by the INCREASE trial, potentially reflecting a unique therapeutic opportunity for this subtype with dose intensification. The number of metastatic organs (≥2) was confirmed as a strong predictor of inferior PFS. These findings support double-dose icotinib as a viable and well-tolerated first-line strategy, especially for L858R-mutant patients and those ineligible for newer-generation TKIs or clinical trials. Future prospective studies in larger cohorts are warranted to confirm the differential impact on mutation subtypes and explore combinatorial approaches for high metastatic burden.

## Data Availability

The original contributions presented in the study are included in the article/supplementary material. Further inquiries can be directed to the corresponding author/s.
